# The Effects of Aging on Sarcoplasmic Reticulum-Related Factors in the Skeletal Muscle of Mice

**DOI:** 10.3390/ijms25042148

**Published:** 2024-02-10

**Authors:** Yuji Kanazawa, Tatsuo Takahashi, Mamoru Nagano, Satoshi Koinuma, Yasufumi Shigeyoshi

**Affiliations:** 1 Department of Physical Therapy, Hokuriku University, Kanazawa 920-1180, Ishikawa, Japan; 2 Department of Anatomy and Neurobiology, Faculty of Medicine, Kindai University, Osakasayama 589-8511, Osaka, Japan; m-nagano@med.kindai.ac.jp (M.N.); koi@med.kindai.ac.jp (S.K.); shigey@med.kindai.ac.jp (Y.S.); 3 Department of Clinical Pharmacology, Hokuriku University, Kanazawa 920-1181, Ishikawa, Japan; t-takahashi@hokuriku-u.ac.jp

**Keywords:** aging, skeletal muscle, sarcoplasmic reticulum, tubular aggregate, slow-twitch muscle, fast-twitch muscle

## Abstract

The pathogenesis of sarcopenia includes the dysfunction of calcium homeostasis associated with the sarcoplasmic reticulum; however, the localization in sarcoplasmic reticulum-related factors and differences by myofiber type remain unclear. Here, we investigated the effects of aging on sarcoplasmic reticulum-related factors in the soleus (slow-twitch) and gastrocnemius (fast-twitch) muscles of 3- and 24-month-old male C57BL/6J mice. There were no notable differences in the skeletal muscle weight of these 3- and 24-month-old mice. The expression of *Atp2a1*, *Atp2a2*, *Sln*, and *Pln* increased with age in the gastrocnemius muscles, but not in the soleus muscles. Subsequently, immunohistochemical analysis revealed ectopic sarcoplasmic reticulum calcium ion ATPase (SERCA) 1 and SERCA2a immunoreactivity only in the gastrocnemius muscles of old mice. Histochemical and transmission electron microscope analysis identified tubular aggregate (TA), an aggregation of the sarcoplasmic reticulum, in the gastrocnemius muscles of old mice. Dihydropyridine receptor α1, ryanodine receptor 1, junctophilin (JPH) 1, and JPH2, which contribute to sarcoplasmic reticulum function, were also localized in or around the TA. Furthermore, JPH1 and JPH2 co-localized with matrix metalloproteinase (MMP) 2 around the TA. These results suggest that sarcoplasmic reticulum-related factors are localized in or around TAs that occur in fast-twitch muscle with aging, but some of them might be degraded by MMP2.

## 1. Introduction

Skeletal muscle accounts for approximately 40% of the total body weight and is mainly composed of muscle fibers [1]. Muscle fibers can contract and relax, and calcium ions play an important role in their contraction–relaxation mechanism [2]. The sarcoplasmic reticulum is involved in the regulation of calcium ion concentrations within muscle fibers [2] and comprises a pouch-like membrane structure surrounding myofibrils [3]. Normally, calcium ions are stored in the sarcoplasmic reticulum, and calcium ion concentrations outside the sarcoplasmic reticulum are kept low, maintaining muscle fibers in a relaxed state [2]. When impulses from neurons reach muscle fibers, dihydropyridine receptor α1 (DHPRα1) in the transverse tubule (t-tubule) receives the signal and changes its conformation, resulting in the release of calcium from ryanodine receptor 1 (RyR1) localized to the terminal cisternae of the sarcoplasmic reticulum [2]. An increase in the calcium concentration in muscle fibers causes muscle contraction [2]. The recovery of calcium ions from the sarcoplasmic reticulum is mediated by the sarcoplasmic reticulum calcium ion ATPase (SERCA), which is present in the sarcoplasmic reticulum membrane [2,3]. In addition, sarcolipin (SLN) and phospholaban (PLN) play important roles in calcium ion homeostasis within muscle fibers as inhibitory regulators of SERCA [4]. Thus, muscle fiber contraction and relaxation properties are supported by sarcoplasmic reticulum-related factors.

Junctophilin (JPH) is also a sarcoplasmic reticulum-related factor, which consists of a family of proteins that link the plasma membrane to the endo/sarcoplasmic reticulum [5]. Four JPH isotypes are primarily expressed in muscle and neuronal cell types [5]. Both JPH1 and JPH2 are expressed in the skeletal muscle, with JPH2 being the major isotype in cardiac and smooth muscle [6,7,8]. Skeletal muscle from neonatal JPH1-knockout mice exhibits an abnormal sarcoplasmic reticulum structure [9]. JPH1-knockout mice also have weak muscle contraction and die within a day after birth owing to lactation defects, suggesting that JPH1 is important for muscle function [9]. Heart-specific knockdown of JPH2 also prevents t-tubule development [10,11]. Furthermore, the knockdown of both JPH1 and JPH2 in mouse skeletal muscle also showed deformation of t-tubule and sarcoplasmic reticulum junctional structures, indicating that calcium homeostasis is impaired [12]. These previous studies show that JPH1 and JPH2 are essential for the maintenance of calcium homeostasis in muscle cells, as they form the junction between the sarcoplasmic reticulum and t-tubules and are responsible for structural stability. Therefore, JPH1 and JPH2 are important factors that support the functionalization of the sarcoplasmic reticulum.

Sarcopenia refers to age-related loss of muscle mass and strength, which subsequently has a negative impact on the health of the elderly [13]. The pathogenesis of sarcopenia involves multiple factors, including chronic inflammatory pathologies and lifestyle-related factors; however, the details remain unclear [14]. Recent studies have reported that the pathogenesis of sarcopenia involves decreased SERCA activity [15]. Decreased calcium uptake by the sarcoplasmic reticulum leads to an increased accumulation of cytosolic calcium ions [16], and higher levels of cytosolic calcium reduce the sensitivity of the contractile apparatus to calcium ions, leading to muscle wasting [17]. However, the effects of aging on the localization of sarcoplasmic reticulum-related factors remain unclear in sarcopenia. A further elucidation of the effects of sarcoplasmic reticulum-related factors on skeletal muscle aging is needed to understand the pathophysiology of sarcopenia and develop therapeutic strategies.

Skeletal muscles are divided into two main groups: slow- and fast-twitch muscles [18]. Slow-twitch muscles are rich in capillaries and mitochondria and have an advantage in terms of aerobic capacity, making them useful as antigravity muscles, such as the soleus muscles [19]. Fast-twitch muscles have larger fiber diameters than slow-twitch muscles, which are advantageous for instantaneous muscle contraction. The gastrocnemius muscles is a typical example of a fast-twitch muscle in the lower leg [19]. Although the soleus and gastrocnemius muscles are located in close proximity, their characteristics and functions differ, and smooth physical movement is achieved when both types of skeletal muscles function. Fast-twitch muscle atrophy is the characteristic pathology of muscle atrophy [20,21,22,23]. However, relative muscle weight loss, loss of stiffness, and loss of the ability to recover occur with aging in slow-twitch muscles as well [24,25]. Therefore, aging has a negative impact on fast- and slow-twitch muscles. However, the molecular mechanisms and localization of sarcoplasmic reticulum-related factors in the pathogenesis of sarcopenia according to muscle fiber type remain unclear. A comparison of the effects of aging on sarcoplasmic reticulum-related factors between slow- and fast-twitch muscles will contribute to the elucidation of the pathology of sarcopenia and provide fundamental data for the consideration of therapeutic strategies. Here, we used soleus and gastrocnemius muscles from young and old mice to analyze the effects of aging on sarcoplasmic reticulum-related factors according to muscle fiber type at the molecular level and protein localization.

## 2. Results

### 2.1. Body Weight, Muscle Weight, and Relative Muscle Weight

**Figure 1:**
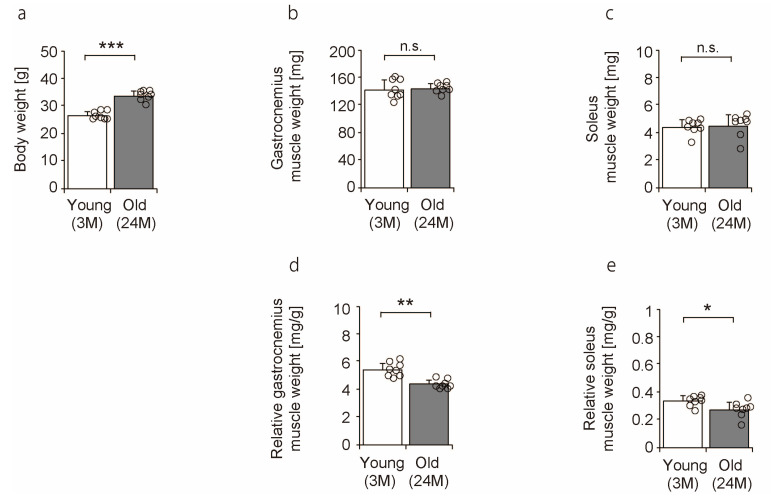
Comparison of body weight, muscle weight, and relative muscle weight between young and old mice. Body weight (**a**), gastrocnemius muscles weight (**b**), soleus muscles weight (**c**), and relative muscle weights of the gastrocnemius (**d**) and soleus (**e**) muscles are shown. Data are presented as mean ± standard deviation (*n* = 8 per group). Significant differences between young and old mice were determined using Student’s *t*-test. *** *p <* 0.0001, ** *p <* 0.001, * *p <* 0.05. n.s.: not significant. All data are represented as dots in the graph. 3M, 3-month-old mice; 24M, 24-month-old mice.

To confirm the effects of aging on skeletal muscle in mice, body weight, muscle weight, and relative muscle weight were measured. Body weight was significantly higher in old mice than in young mice (Figure 1a). There was no significant difference in the gastrocnemius and soleus muscles between young and old mice (Figure 1b,c). However, a significant decrease was observed in the relative weights of both the gastrocnemius and soleus muscles with age (Figure 1d,e). Previous studies have shown that the skeletal muscle mass of 24-month-old C57BL/6J mice is significantly lower than that of 6- or 15-month-old mice [26,27]. Other studies have also demonstrated that relative muscle weight decreases with age [14,24]. Thus, these results suggest that old mice tend to gain more fat than muscle during the aging process; however, these mice do not lose more muscle, as determined by comparing 3- and 24-month-old mice in this study.

### 2.2. SERCA-Related Factors

**Figure 2:**
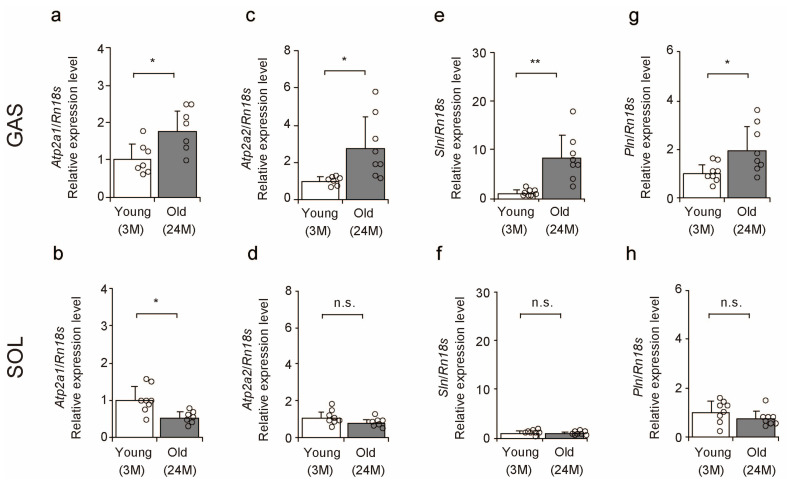
Comparison of the expression levels of sarcoplasmic reticulum calcium ion ATPase-related factors in skeletal muscle. Relative mRNA expression level of *Atp2a1* (**a**,**b**), *Atp2a2* (**c**,**d**), *Sln* (**e**,**f**), and *Pln* (**g**,**h**) in the gastrocnemius and soleus muscles. Data are presented as mean ± standard deviation, *n* = 7–8 per group. Significant differences between young and old mice were determined using Student’s *t*-test. ** *p <* 0.001, * *p <* 0.05. n.s.: not significant. All data are represented as dots in the graph. GAS, gastrocnemius muscle; SOL, soleus muscle; 3M, 3-month-old mice; 24M, 24-month-old mice.

To confirm the effects of aging on the gene expression levels of SERCA and its inhibitors, we used a quantitative polymerase chain reaction (qPCR) to determine the expression levels of *Atp2a1* and *Atp2a2*, which encode the SERCA1 and SERCA2a proteins [28,29,30], and *Sln* and *Pln*, which inhibit calcium ion reabsorption in the sarcoplasmic reticulum [3]. In the gastrocnemius muscles, *Atp2a1* and *Atp2a2* expression increased with age (Figure 2a,c). In the soleus muscles, *Atp2a1* expression decreased with age (Figure 2b), whereas there was no significant difference in *Atp2a2* expression in the soleus muscles (Figure 2d). *Sln* and *Pln* expression increased with age in the gastrocnemius muscles (Figure 2e,g) but did not change in the soleus muscles (Figure 2f,h). These results suggest that the effects of aging on the expression of SERCA-related factors vary between fast- and slow-twitch muscles.

### 2.3. SERCA Localization 

**Figure 3:**
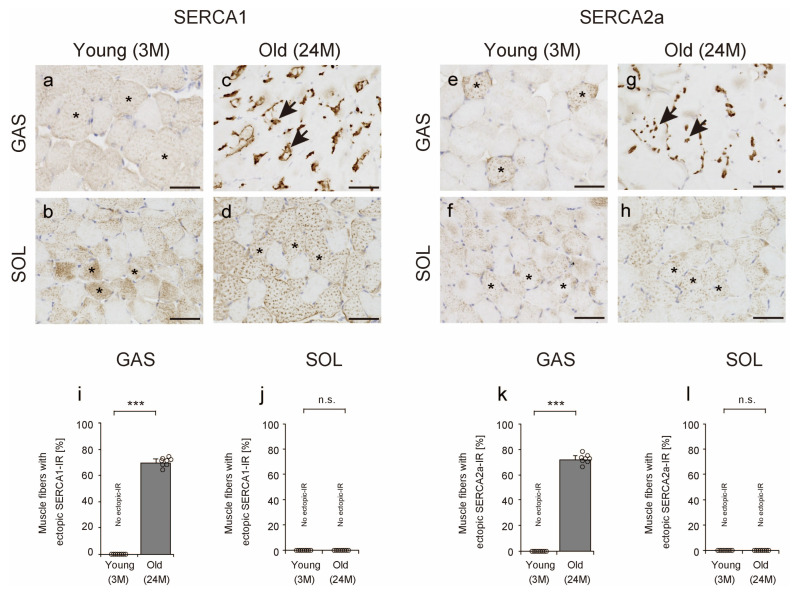
Sarcoplasmic reticulum calcium ion ATPase (SERCA) localization. Cross-sections of the gastrocnemius (**a**,**c**,**e**,**g**) and soleus (**b**,**d**,**f**,**h**) muscles were stained with anti-SERCA1 (**a**–**d**) or anti-SERCA2a (**e**–**h**) antibodies. Asterisks indicate normal immunoreactivity (IR) of SERCA1 or SERCA2a in the cytoplasm (**a**,**b**,**d**–**f**,**h**). Arrows indicate muscle fiber with ectopic IR of SERCA1 or SERCA2a (**c**,**g**). Scale bar = 50 μm. The number of muscle fibers with ectopic SERCA1 or SERCA2a IR was measured in the gastrocnemius (**i**,**k**) and soleus (**j**,**l**) muscles. Data are presented as mean ± standard deviation, *n* = 8 per group. Significant differences between young and old mice were determined using Student’s *t*-test. *** *p* < 0.0001. n.s.: not significant. All data are represented as dots in the graph. GAS, gastrocnemius muscle; SOL, soleus muscle; 3M, 3-month-old mice; 24M, 24-month-old mice.

To confirm the effects of aging on SERCA1 and SERCA2a, we assessed their localization using immunohistochemical analysis (IHC). In young mice, SERCA1 and SERCA2a localized to the cytoplasmic region of the muscle fiber in the transverse section (asterisks in Figure 3a,b,e,f). In contrast, in the gastrocnemius muscles of old mice, localization of SERCA1 and SERCA2a was presented as stains indicating high immunoreactivity (IR) in the central or subsarcolemmal region of muscle fibers (arrows in Figure 3c,g). Such ectopic IR was observed in numerous muscle fibers of the gastrocnemius muscles in old mice. In addition, ectopic IR was scarce in the soleus muscles of the old mice (Figure 3d,h). In fact, the number of fibers with ectopic IR indicating the localization of SERCA1 or SERCA2a markedly increased in the gastrocnemius muscles with age (Figure 3i,k), but such changes were not observed in the soleus muscles in this study (Figure 3j,l). These results suggest that SERCA shows ectopic IR in fast-twitch muscles with age.

### 2.4. Histochemical Findings 

**Figure 4:**
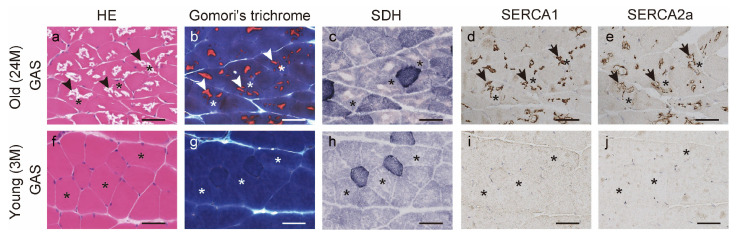
Histochemical findings and ectopic sarcoplasmic reticulum calcium ion ATPase (SERCA) immunoreactivity (IR). Serial cross-sections of the gastrocnemius muscles of old (**a**–**c**) and young mice (**f**–**h**) were stained with hematoxylin and eosin (HE), modified Gomori’s trichrome, and succinate dehydrogenase (SDH). These cross-sections were also stained with anti-SERCA1 or anti-SERCA2a antibodies in old (**d**,**e**) and young mice (**i**,**j**). Black arrowheads indicate spikes and lakes in the cytoplasm (**a**). White arrowheads indicate red materials in cytoplasm (**b**). Black arrows indicate ectopic SERCA IR (**d**,**e**). Asterisks denote identical muscle fibers. Scale bar = 50 μm. GAS, gastrocnemius muscle; 3M, 3-month-old mice; 24M, 24-month-old mice.

To confirm the pathological findings indicating ectopic-SERCA IR, skeletal muscle was also subjected to routine histochemical staining of hematoxylin and eosin (HE), modified Gomori’s trichrome, and succinate dehydrogenase (SDH) staining. In the gastrocnemius muscles of old mice, numerous splits and lakes were formed in the cytoplasm, and clear or pink material was observed inside the splits and lakes after HE staining (black arrowheads in Figure 4a). Following Gomori’s trichrome staining, red material was observed in the cytoplasm (white arrowheads in Figure 4b), suggesting the localization of special structures in the muscle fibers. However, after staining with SDH, a mitochondrial stain, the HE-stained areas with splits and lakes and Gomori’s trichrome-stained red materials reduced slightly, but no other clear pathological findings were observed (Figure 4c). These pathological findings indicate the abnormal accumulation of the sarcoplasmic reticulum, which shows tubular aggregates (TAs) [31,32], and suggest that TAs form in the gastrocnemius muscles of old mice. The above findings were not observed in the soleus and gastrocnemius muscles of the young mice or in the soleus muscles of the old mice in this study (Figure 4f–h). In addition, ectopic SERCA1 and SERCA2a IR co-localized with HE-stained splits and lakes and Gomori’s trichrome-stained red materials (Figure 4a,b,d,e). These results suggest that ectopic-SERCA IR can reflect TA.

### 2.5. Electron Microscopic Analysis

**Figure 5:**
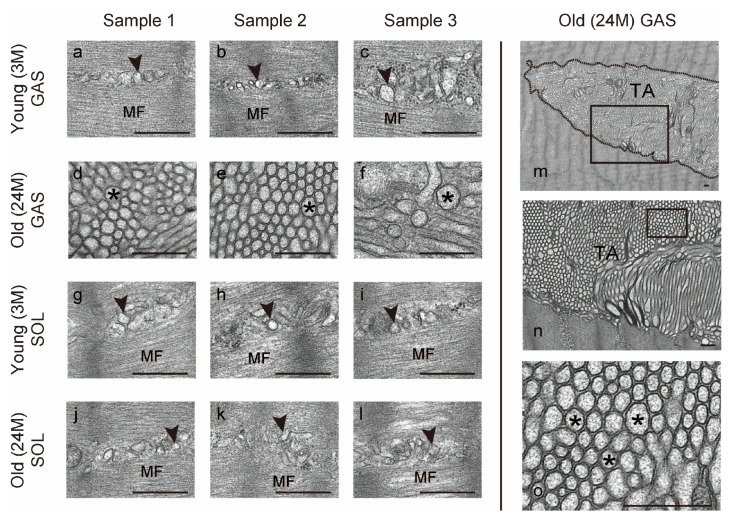
Electron microscopic analysis. Ultrathin longitudinal sections of gastrocnemius (**a**–**f**) and soleus muscles (**g**–**l**) from the young and old mice were examined using transmission electron microscopy (*n* = 3 per group). Arrowheads: sarcoplasmic reticulum; asterisks: representative circular cross section of tubular aggregate (TA); inside the dotted line: TA; MF, myofibrils. Parts (**n**,**o**) are enlargements of the rectangular regions in (**m**,**n**). Scale bar = 500 nm. GAS, gastrocnemius muscle; SOL, soleus muscle; 3M, 3-month-old mice; 24M, 24-month-old mice.

To confirm the presence of TAs, the sarcoplasmic reticulum structure was observed using a transmission electron microscope (TEM). The sarcoplasmic reticulum of muscle fibers is a convoluted structure composed of various tubules and cisternae, which branch to form a network that surrounds each myofibril [33]. Representative circular cross-sections of the sarcoplasmic reticulum of young and old soleus muscles and young gastrocnemius muscles were observed in TEM (arrowheads in Figure 5a–c,g–l). Furthermore, TAs were observed in the cytoplasm in the old gastrocnemius muscles (inside the dotted line in Figure 5m). The cross-section of the TA was observed as an aggregation of structures with various shapes including circular and tubular shapes (Figure 5l). Representative circular sections are shown (asterisks in Figure 5d–f,n). In this study, we were unable to observe TAs in young and old soleus muscles and young gastrocnemius muscles using TEM. These results confirm that TAs, an abnormal accumulation of the sarcoplasmic reticulum, occur in aged gastrocnemius muscles.

### 2.6. DHPRα1 and RyR1 Localization

**Figure 6:**
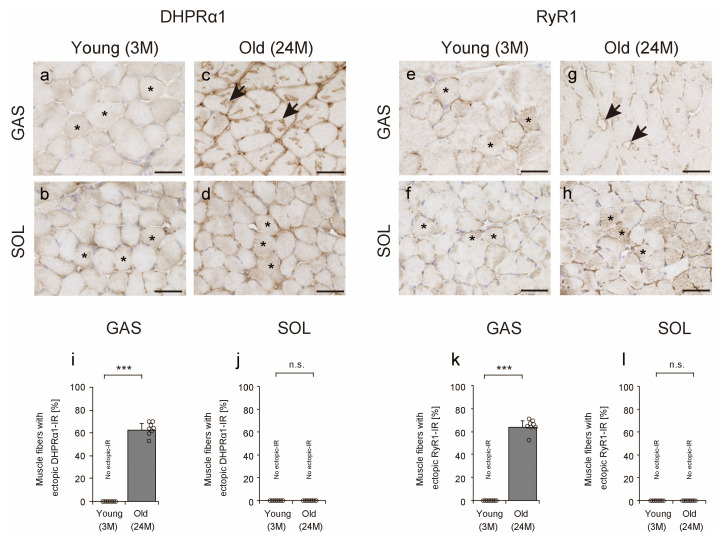
Dihydropyridine receptor alpha 1 (DHPRα1) and ryanodine receptor 1 (RyR1) localization. Cross-sections of the gastrocnemius (**a**,**c**,**e**,**g**) and soleus (**b**,**d**,**f**,**h**) muscles were stained with anti-DHPRα1 (**a**–**d**) or anti-RyR1 (**e**–**h**) antibodies. Asterisks indicate normal immunoreactivity (IR) in the cytoplasm and subsarcolemma of DHPRα1 or RyR1 (**a**,**b**,**d**–**f**,**h**). Arrows indicate muscle fiber with ectopic DHPRα1 or RyR1 IR (**c**,**g**). Scale bar = 50 μm. The number of muscle fibers with ectopic DHPRα1 or RyR1 IR was measured in the gastrocnemius (**i**,**k**) and soleus (**j**,**l**) muscles. Data are presented as mean ± standard deviation, *n* = 8 per group. Significant differences between young and old mice were determined using Student’s *t*-test. *** *p* < 0.0001. n.s.: not significant. All data are represented as dots in the graph. GAS, gastrocnemius muscle; SOL, soleus muscle; 3M, 3-month-old mice; 24M, 24-month-old mice.

The presence of DHPRα1 localized to the t-tubule and RyR1 localized to the terminal cisternae of the sarcoplasmic reticulum are essential for the function of the sarcoplasmic reticulum [34]. To confirm whether factors that contribute to the function of the sarcoplasmic reticulum are also localized in the TA, we observed the localization of DHPRα1 and RyR1 in skeletal muscle via IHC. In the young and old soleus muscles and young gastrocnemius muscles, DHPRα1 and RyR1 localizations were observed in the cytoplasm and subsarcolemma (asterisks in Figure 6a,b,d–f,h). Conversely, in old gastrocnemius muscles, DHPRα1 localization was observed in the TA and RyR1 localization was observed in the contours of the TA (arrows in Figure 6c,g). The number of muscle fibers with ectopic DHPRα1 or RyR1 IR, which indicates the localization in or around the TA, increased markedly in the gastrocnemius muscles of old mice (Figure 6i,k), but no such changes were observed in the soleus muscles (Figure 6j,l). These results suggest that DHPRα1 localizes in the TA and RyR1 localizes to the TA’s periphery.

### 2.7. JPH1 and JPH2 Localization

**Figure 7:**
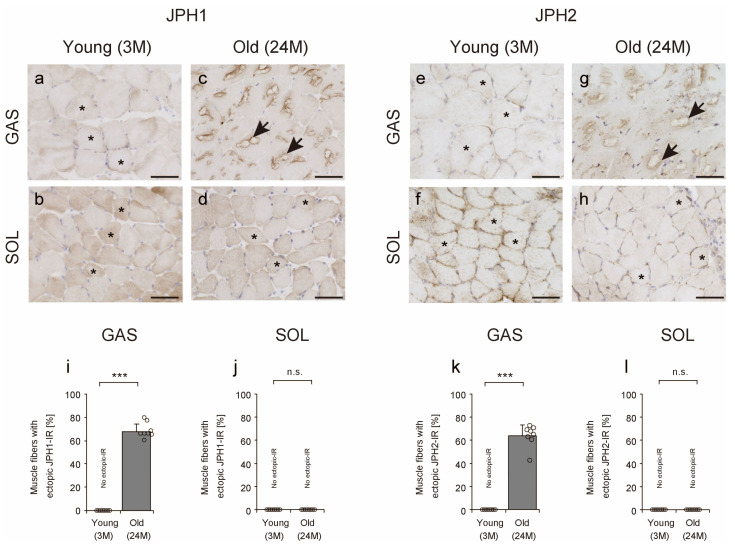
Junctophilin (JPH) 1 and JPH2 localization. Cross-sections of the gastrocnemius (**a**,**c**,**e**,**g**) and soleus (**b**,**d**,**f**,**h**) muscles were stained with anti-JPH1 (**a**–**d**) or anti-JPH2 (**e**–**h**) antibodies. Asterisks indicate normal immunoreactivity (IR) to JPH1 or JPH2 in the cytoplasm and subsarcolemma (**a**,**b**,**d**–**f**,**h**). Arrows indicate muscle fiber with ectopic JPH1 or JPH2 IR (**c**,**g**). Scale bar = 50 μm. The number of muscle fibers with ectopic JPH1 or JPH2 IR was measured in the gastrocnemius (**i**,**k**) and soleus (**j**,**l**) muscles. Data are presented as mean ± standard deviation, *n* = 8 per group. Significant differences between young and old mice were determined using Student’s *t*-test. *** *p* < 0.0001. n.s.: not significant. All data are represented as dots in the graph. GAS, gastrocnemius muscle; SOL, soleus muscle; 3M, 3-month-old mice; 24M, 24-month-old mice.

JPH connects and stabilizes the sarcoplasmic reticulum and plasma membrane and is essential for the physical interaction of DHPRα1 and RyR1. Moreover, JPH1 localizes to skeletal muscle, while JPH2 localizes to all muscle tissues, including cardiac, skeletal, and smooth muscle tissues [5]. In this study, we used IHC to investigate whether JPH1 and JPH2 are also localized in the TA. First, JPH in young and old soleus muscles and young gastrocnemius muscles were identified in both the cytoplasm and subsarcolemma (asterisks in Figure 7a,b,d–f,h). In particular, JPH1 staining was slightly stronger in the cytoplasm than JPH2 staining was, reflecting the higher abundance of JPH1 than of JPH2 in skeletal muscle [5]. Second, in old gastrocnemius muscles, JPH1 and JPH2 localization was observed ectopically in the contours of the TA (arrows in Figure 7c,g). The number of muscle fibers with ectopic JPH1 and JPH2 IR increased markedly in the gastrocnemius muscles of old mice (Figure 7i,k), but no such changes were observed in the soleus muscles (Figure 7j,l). These results suggest that JPH1 and JPH2 are localized around the TA.

### 2.8. Matrix Metalloproteinase 2 Localization

**Figure 8:**
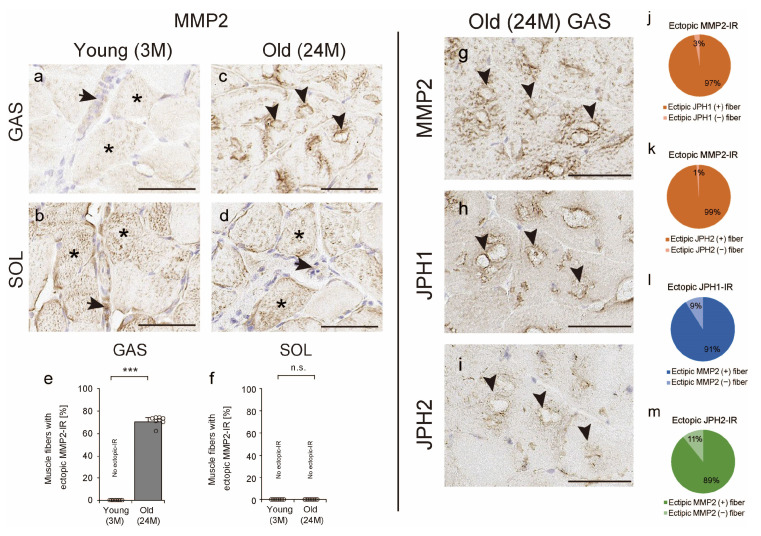
Matrix metalloproteinase 2 (MMP2) and junctophilin (JPH). Cross-sections of the soleus and gastrocnemius muscles in the young mice (**a**,**b**) and old mice (**c**,**d**) were stained with MMP2 antibody. Asterisks and arrows indicate immunoreactivity (IR) to MMP2 in the cytoplasm and extracellular matrix (**a**,**b**,**d**). Arrowheads indicate muscle fiber with ectopic MMP2 IR (**c**). The number of muscle fibers with ectopic MMP2 IR was measured in the gastrocnemius (**e**) and soleus (**f**) muscles. Arrows in serial cross-sections (**g**–**i**) indicate MMP2 (**g**) localized to the contour of the TA and co-localization with JPH1 (**h**) and JPH2 (**i**) in the gastrocnemius muscles of old mice. The concordance (ectopic JPH1 IR/ectopic MMP2 IR) and concordance (ectopic JPH2 IR/ectopic MMP2 IR) rates (**j**,**k**) as well as the concordance (ectopic MMP2 IR/ectopic JPH1 IR) and concordance (ectopic MMP2 IR/ectopic JPH2 IR) rates (**l**,**m**) were measured in old gastrocnemius muscles. Data are presented as mean ± standard deviation, *n* = 8 per group. Significant differences between young and old mice were determined using Student’s *t*-test. *** *p* < 0.0001. n.s.: not significant. All data are represented as dots in the graph. Scale bar = 50 μm. GAS, gastrocnemius muscle; SOL, soleus muscle; 3M, 3-month-old mice; 24M, 24-month-old mice.

JPH is susceptible to degradation as a substrate for matrix metalloproteinase 2 (MMP2) [35]. In this study, the localization of JPH1, JPH2, and MMP2 in the TA was confirmed via IHC. First, in the young and old soleus muscles and young gastrocnemius muscles, MMP2 localization was observed in the cytoplasm (asterisks in Figure 8a,b,d) and extracellular matrix (arrows in Figure 8a,b,d). In contrast, in the old gastrocnemius muscles, MMP2 localization was observed ectopically in the contour of the TA (arrowheads in Figure 8c). The number of muscle fibers with ectopic MMP2 IR increased markedly in the gastrocnemius muscles of old mice (Figure 8e), but no such changes were observed in the soleus muscles (Figure 8f). In addition, JPH1 or JPH2 were, respectively, co-localized with MMP2 around the contours of the TA (Figure 8g–i). In fact, the concordance (ectopic JPH1 IR/ectopic MMP2 IR) and concordance (ectopic JPH2 IR/ectopic MMP2 IR) rates were 97% and 99% (Figure 8j,k). The concordance (ectopic MMP2 IR/ectopic JPH1 IR) and concordance (ectopic MMP2 IR/ectopic JPH2 IR) rates were 91% and 89% (Figure 8l,m). These results suggest that JPH1 or JPH2 co-localize with MMP2 around the TA.

### 2.9. Ratio of Type I and II Fibers

**Figure 9:**
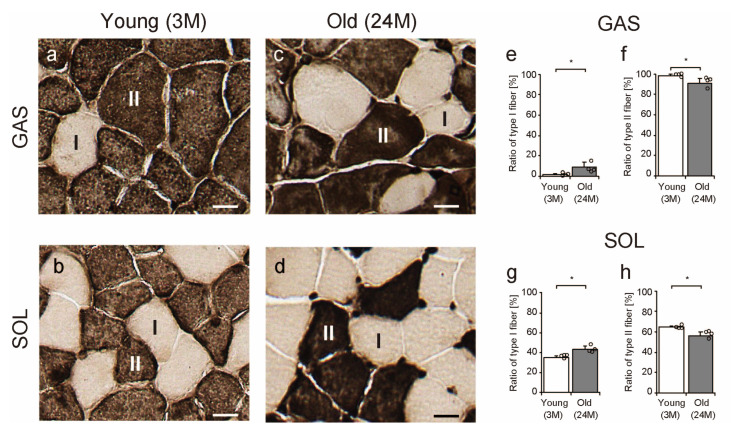
Ratio of type I and II fibers. Cross-sections of the gastrocnemius and soleus muscles were stained with myofibrillar adenosine triphosphatase in young (**a**,**b**) and old (**c**,**d**) mice. Lightly and darkly stained muscle fibers were classified as type I and type II muscle fibers, respectively. Scale bar = 10 μm. The ratios of type I and II muscle fiber were measured in the gastrocnemius (**e**,**f**) and soleus (**g**,**h**) muscles. Data are presented as mean ± standard deviation, *n* = 4 per group. Significant differences between young and old mice were determined using Student’s *t*-test. * *p* < 0.05. All data are represented as dots in the graph. GAS, gastrocnemius muscle; SOL, soleus muscle; 3M, 3-month-old mice; 24M, 24-month-old mice.

To confirm the ratio of type I (slow-twitch) and type II (fast-twitch) muscle fibers, sections were stained with myofibrillar adenosine triphosphatase (mATPase) and observed (Figure 9a–d). Type II fibers were more abundant in the gastrocnemius muscles than in the soleus muscles, while type I fibers were more abundant in the soleus muscles than in the gastrocnemius muscles in both young and old mice (Figure 9a–d). With age, the ratio of type II fibers decreased, while that of type I fibers increased in the gastrocnemius and soleus muscles (Figure 9e–h).

### 2.10. DHPRα1 and SERCA1 Co-Localization

**Figure 10:**
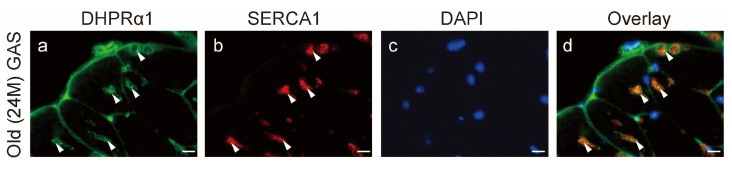
DHPRα1 and SERCA1 co-localization. Cross sections of the old gastrocnemius muscles were stained by anti-DHPRα1 (**a**) and SERCA1 (**b**) antibodies. DAPI was used for counterstaining (**c**). Arrowheads indicate representative localizations of DHPRa1 (**a**) and SERCA1 (**b**) in tubular aggregates (TAs), and arrowheads of overlay image shows representative co-localizations of DHPRa1 and SERCA1 in TAs (**d**). Scale bar = 10 μm. GAS, gastrocnemius muscle; 24M, 24-month-old mice.

To confirm the co-localization of sarcoplasmic reticulum-related factors in TAs, we analyzed the localization of DHPRα1 and SERCA1 via immunofluorescence staining (Figure 10a–d). According to the optical microscopic observations, DHPRα1 and SERCA1 were co-localized in the TA (Figure 10d). These results support the localization of sarcoplasmic reticulum-related factors in the TA.

## 3. Discussion

In this study, we examined the effects of aging on sarcoplasmic reticulum-related factors in mouse skeletal muscle. First, qPCR results showed that the expressions of *Atp2a1*, *Atp2a2*, *Sln*, and *Pln* increased with age in fast-twitch muscle, but not in slow-twitch muscle. Subsequently, IHC showed ectopic SERCA1 and SERCA2a IR only in the gastrocnemius muscles of the old mice. Histochemical and TEM analyses identified TAs in the gastrocnemius muscles of old mice, suggesting that ectopic SERCA IR reflects the presence of TAs. In addition, DHPRα1, RyR1, JPH1, and JPH2, which contribute to the function of the sarcoplasmic reticulum, also localize in or around the TA according to our IHC findings. Interestingly, JPH1 and JPH2 were found to co-localize with MMP2 around the TA. These results suggest that the sarcoplasmic reticulum as well as functionalizing factors localize in or around TAs generated in fast-twitch muscle during aging, but that JPH, a functionalizing factor, may be degraded by MMP2 around the TA.

Increased expression of *Atp2a1*, *Atp2a2*, *Sln*, and *Pln* with age may reflect a compensatory response of fast-twitch muscle with regard to aging. *Atp2a1* encodes the SERCA1 isoform expressed in adult fast-twitch muscle, and *Atp2a2* encodes the SERCA2a isoform common to cardiac and slow-twitch muscle [28,29,30]. Similar to this study, *Atp2a2* expression was reported to be increased in aged fast-twitch muscle [36], which is thought to be an aging phenomenon where fast-twitch muscle features are no longer retained and transition to slow-twitch muscles. In this study, the reduction in *Atp2a1* expression in the soleus muscles may reflect the loss of fast-twitch muscle fibers with age. However, the variation in *Atp2a1* and *Atp2a2* observed in this study could not be explained solely by age-related muscle fiber type transitions. In fact, *Atp2a1* expression in fast-twitch muscles also increased in the gastrocnemius muscles with age. A previous study suggested that variations in SERCA-related factors not only reflect muscle fiber type transition, but also reflect compensatory responses to impaired calcium ion homeostasis [28]. Furthermore, SLN and PLN, which inhibit the calcium transport properties of SERCA [4], are upregulated in various types of muscular dystrophies [37] and after denervation [38]. However, the transgenic overexpression of SLN in skeletal muscle is not harmful and can promote oxidative metabolism and exercise capacity [39,40]. According to the results of previous studies and this study, a compensatory response to meet the impaired calcium ion homeostasis and high energy demand associated with aging may occur in aged fast-twitch muscles.

Functionalizing factors of the sarcoplasmic reticulum can be localized in the TA or around the TA but might not be fully functional. In this study, ectopic IR to SERCA1, SERCA2a, DHPRα1, RyR1, JPH1, and JPH2 were identified in or around the TA. In particular, SERCA1, SERCA2a, and DHPRα1 showed relatively strong IR in the TA, and RyR1 was found in the contour and periphery of the TA. These findings were also confirmed to be specific to fast-twitch muscles in this study. These results are consistent with those of previous studies [31,41,42], which corroborated the multi-protein confirmation of TAs in aged fast-twitch muscle in this study. Furthermore, this study revealed that JPH1 and JPH2 localize to the contour and periphery of the TA. JPH1 and JPH2 physically connect the plasma membrane to the sarcoplasmic reticulum and support the interaction between DHPRα1 and RyR1 [5]. Therefore, these results suggest that the TA contour contains functional factors required for calcium ion homeostasis by the sarcoplasmic reticulum. Furthermore, the destabilization of the linkage between the plasma membrane and endoplasmic reticulum by JPH has been hypothesized to be involved in TA formation [43]. The hypothesis and results of this study suggest that JPH1 and JPH2 localized around the TA may not adequately link the plasma membrane to the sarcoplasmic reticulum.

JPH1 and JPH2 localized in and around the TA contour might be subject to degradation. In this study, we found that JPH1 or JPH2 co-localizes with MMP2 in and around the TA contour. Previous studies have reported that MMP2 co-localizes with and further degrades JPH2 protein in the pathogenesis of myocardial injury owing to post-ischemic reperfusion [35]. Therefore, the co-localization of JPH1 or JPH2 with MMP2 in and around the TA contour might indicate that JPH is a target for MMP2-induced degradation. JPH1 and JPH2 are essential for normal skeletal and cardiac muscle function, as proved by the fact that JPH1- or JPH2-knockout animals die immediately after birth or are embryonically lethal [5]. Conversely, JPH1 overexpression results in the formation of abnormal stacks of endoplasmic reticulum cisternae [7]. Heart-specific JPH2 overexpression in mice increased endoplasmic reticulum and plasma membrane contact site area [44]. Furthermore, artificial expression of several endoplasmic reticulum proteins has been reported to result in the accumulation of endoplasmic reticulum membranes [45,46]. Therefore, the expression of JPH1 and JPH2 around the TA might be conducive to TA formation, and MMP2 expression might reflect TA removal response via JPH degradation. In a previous study, TAs segregate SERCA away from the myofibrils and contain SERCA that is likely blocked in a nonfunctional configuration, suggesting that the TA is not actively involved in calcium ion homeostasis of muscle fibers [41]. Interestingly, voluntary exercise habits decrease TA and improve calcium homeostasis and muscle function [16,47]. Furthermore, skeletal muscle exercises such as resistance training and stretching are known to increase MMP2 activity and expression [48,49]. These results show that the degradation of the TA associated with aging could improve muscle function, and MMPs may be involved in this degradation. MMP2 may be important in the removal of the TA via JPH degradation, and the relationship between TA and MMP2 is worth researching further in the future.

Although interesting findings were obtained, this study has several limitations. First, this study showed that JPH1 or JPH2 and MMP2 were co-expressed around the TA, but it is unclear whether JPH or MMP2 was formed earlier or at the same time. Previous studies that followed the origins of the TA over time in aging mice reported the gradual, stepwise generation of the TA [41]. Therefore, in the future, understanding the localization of JPH and MMP2 at the time when the TA begins to develop may provide a better understanding of the significance of JPH and MMP2 in the TA. Second, detailed analysis by muscle fiber type was not available for the TA and sarcoplasmic reticulum-related factors in this study. For convenience, the gastrocnemius and soleus muscles were used as the fast-twitch and slow-twitch muscles, respectively, in this study. However, the gastrocnemius muscles also contains slow-twitch muscle fibers, while the soleus muscles also contains fast-twitch muscle fibers (Figure 9). In addition, aging alters the ratio of muscle fiber types characterized by a decrease in type II (fast-twitch) fibers (Figure 9). In this study, the TA was identified only in the gastrocnemius muscles of old mice. The gastrocnemius muscles has an abundance of type IIB fibers, which are not present in the soleus muscles [50]. A previous study has also reported that the TA is a type IIB fiber-specific finding [51]. Therefore, TA aggregation is thought to occur in type IIB fibers of old mice in this study. In addition, we believe that a further understanding about muscle fibers can be achieved by combining detailed muscle-fiber typing with an analysis of sarcoplasmic reticulum-associated factors in the future. Third, an unusual method of TEM sample preparation was chosen for this study in order to obtain valuable old mice muscle samples for multiple molecular biological and morphological experiments. Frozen sections also allow for precise sectioning into cross-sectional and longitudinal sections. Sarcoplasmic reticula and TAs were visible. However, a further consideration must be given to the sample preparation process to analyze the microstructure of the sarcoplasmic reticulum in greater detail. Fourth, this study could not directly assess calcium ion utilization in the soleus and gastrocnemius muscles. A direct measurement of calcium homeostasis involving sarcoplasmic reticulum-related factors is important for understanding muscle function better. Fifth, we could not demonstrate the functional significance of the TA in aged fast-twitch muscle. A previous study has shown that long-term exercise habits prevent TA formation and restore fatigue tolerance [47]. Therefore, the TA may influence fatigue tolerance in skeletal muscle. We believe that future evaluation of muscle functions, such as muscle strength and muscle endurance, will provide a better understanding of the impact of the TA on muscle function. Sixth, there were no significant differences in skeletal muscle weight between 3- and 24-month-old mice in this study. Future studies need to assess aging progressively to better understand the relationship between sarcopenia and sarcoplasmic reticulum-related factors.

## 4. Materials and Methods

### 4.1. Animals 

Three- and twenty-four-month-old C57BL/6J male mice (*n* = 16) were obtained from the Jackson Laboratory (Yokohama, Japan). After the mice were purchased, they were acclimated for at least one week before being used as experimental animals. The mice were divided into two groups: young (3-month-old mice) and old (24-month-old mice).

All of the animals were housed in individual cages and allowed ad libitum access to food and water. The environmental conditions were maintained at 23 ± 2 °C under a 12:12 h light/dark cycle. This study was approved by the Committee of Animal Care and Use of Hokuriku University (approval number: 23–14; approval date: 10 April 2023). All experimental procedures were conducted in accordance with the institutional guidelines for the use of experimental animals.

### 4.2. Sampling 

The mice were weighed and euthanized by cervical dislocation, and the gastrocnemius and soleus muscles were removed and weighed. The right and left muscles were used for molecular and morphological analyses, respectively. Part of the right lateral head of the gastrocnemius muscle and the entire right soleus muscle were preserved in RNA later (Thermo Fisher Scientific, Waltham, MA, USA), while other parts of the muscles were frozen immediately in isopentane, cooled in dry ice, and stored at −80 °C for further analyses.

### 4.3. qPCR

Total RNA was extracted from the lateral head of the gastrocnemius and soleus muscles using TRIzol reagent (Thermo Fisher Scientific, Waltham, MA, USA). After quality confirmation, the total RNA concentration was normalized to 1 μg and reverse-transcribed to generate first-strand cDNA using random primers and ReverTraAce (Toyobo, Osaka, Japan). qPCR was performed on a CFX96 Touch Real-Time PCR Detection System (Bio-Rad Laboratories, Hercules, CA, USA) using TB Green Premix Ex Taq II (Takara Bio, Kusatsu, Shiga, Japan). The reaction procedure was as follows: 1 cycle at 95 °C for 30 s, followed by 40 cycles at 95 °C for 5 s and 60 °C for 30 s. A calibration curve was created using the template and the expression level of each target gene was normalized to that of the housekeeping gene 18S ribosomal RNA (*Rn18s*). The expression (upregulation or downregulation) of the target genes in the old group was compared with that in the young group. The primers used for qPCR were as follows: 

*Atp2a1*, 5′-ACACAGACCCTGTCCCTGAC-3′ (Forward) and 5′-TGCAGTGGAGTCTTGTCCTG-3′ (Reverse); 

*Atp2a2*, 5′-TCGACCAGTCAATTCTTACAGG-3′ (Forward) and 5′-CAGGGACAGGGTCAGTATGC-3′ (Reverse); 

*Sln*, 5′-GCTCCTCTTCAGGAAGTGAAG-3′ (Forward) and 5′-TGGCCCCTCAGTATTGGTAGG-3′ (Reverse); 

*Pln*, 5′-ATTTCGCCTCCTTACCTCCA-3′ (Forward) and 5′-AGCTTCAGCGTCACGTTTCT-3′ (Reverse); 

*Rn18s*, 5′-GCAATTATTCCCCATGAACG-3′ (Forward) and 5′-GGCCTCACTAAACCATCCAA-3′ (Reverse).

### 4.4. IHC 

Transverse tissue sections (10 µm thickness) were cut from the middle part of the lateral head of the gastrocnemius and soleus muscles using a cryostat (CM1950; Leica, Wetzlar, Germany) at −25 °C and mounted on amino silane-coated glass slides. Subsequently, the transverse sections (10 µm thickness) were fixed in 4% paraformaldehyde and rinsed with phosphate-buffered saline (PBS; pH 7.4). The sections were then bleached with 3% H_2_O_2_, rinsed with PBS, and incubated in PBS containing 1% normal goat serum and 0.3% Triton X-100 at 4 °C for 1 h. The sections were then incubated with mouse monoclonal anti-DHPRα1 antibodies (MA3-920; Thermo Fisher Scientific, Waltham, MA, USA; 1:100) or rabbit polyclonal anti-SERCA1 antibodies (22361-1-AP; Proteintech, Rosemont, IL, USA; 1:500), anti-SERCA2 antibodies (ab3625; Abcam, Cambridge, MA, USA; 1:500), anti-RyR1 antibodies (D4E1, #8153; Cell Signaling Technology, Danvers, MA, USA; 1:100), anti-JPH1 antibodies (40-5100; Thermo Fisher Scientific, Waltham, MA, USA; 1:250), anti-JPH2 antibodies (40-5300; Thermo Fisher Scientific, Waltham, MA, USA; 1:50), or anti-MMP2 antibodies (GTX104577; GeneTex, Irvine, CA, USA; 1:50) in PBS containing 0.3% Triton X-100 at 4 °C for 24 h. Subsequently, the sections were incubated with biotinylated anti-mouse or rabbit immunoglobulin G (Vectastain ABC kit; Vector Laboratories, Burlingame, CA, USA) diluted at 1:1000 in PBS for 1 h at 25 °C, followed by incubation with avidin-biotin complex (Vectastain ABC kit) for 1 h at 4 °C. After rinsing with PBS, the sections were washed with Tris-HCl buffer (pH 7.4) and incubated with diaminobenzidine (0.035%) in Tris-HCl buffer (0.001% H_2_O_2_) for 15 min at 25 °C. After the diaminobenzidine reaction, sections were stained with hematoxylin, dehydrated using a graded series of ethanol rinses, immersed in xylene, and embedded in Permount Mounting Medium (Falma Inc., Tokyo, Japan). To prepare a negative control for IHC, sections were incubated with PBS instead of primary antibodies, and the results showed no staining.

### 4.5. Morphological Analysis

Sections stained with anti-SERCA1, SERCA2a, DHPRα1, RyR1, JPH1, JPH2, or MMP2 antibodies were observed using a microscope (BZ-X800; Keyence, Osaka, Japan). The number of muscle fibers with ectopic IR was determined, and the value was calculated as a percentage of the total number of muscle fibers used for analysis; the area per image was 393,880 μm^2^; three images per gastrocnemius muscles and two images per soleus muscles were analyzed. The ectopic co-localization of JPH1 or JPH2 and MMP2 in the old gastrocnemius muscles was calculated using 3 images of each stain in serial cross-sections to calculate the concordance rate.

### 4.6. Histochemical Analysis 

Serial transverse sections (10 µm thickness) of the gastrocnemius and the soleus muscles were observed with HE, modified Gomori’s trichrome, and SDH staining. In HE staining, nucleus was stained with hematoxylin for 10 min, then with eosin for 1 min. Gomori’s trichrome staining was performed according to the instructions given with the staining kit (TRG-1, ScyTek Laboratories, Logan, UT, USA). In SDH staining, sections were incubated in 0.2 M phosphate buffer (pH 7.6) containing 0.2 M sodium succinate and 0.05% nitroblue tetrazolium for 45 min at 37 °C in accordance with the procedure described in previous studies [52,53]. After staining, sections were dehydrated using a graded series of ethanol rinses, immersed in xylene, and embedded in Permount Mounting Medium (Falma Inc., Tokyo, Japan). Then, stained sections were observed using a microscope (BZ-X800; Keyence, Osaka, Japan).

### 4.7. TEM 

Longitudinal tissue sections (1 mm thickness) were cut from the middle part of the lateral gastrocnemius and soleus muscles’ belly at −25 °C. The sections were fixed with 4% paraformaldehyde/2% glutaraldehyde at 4 °C for 24 h and then treated using osmium tetroxide, after which they were dehydrated using a series of ethanol gradients [25]. Finally, the sections were embedded in Epon. Ultrathin sections (90 nm thickness) were cut using an ultra-microtome and stained with 4% uranyl acetate and 1% lead citrate. Sections were observed using a TEM (HT7700; Hitachi, Tokyo, Japan). The sample size was set to 3 for each group, for a total of 12 samples.

### 4.8. mATPase Staining

Transverse tissue sections (10 µm thickness) were cut from the middle part of the lateral head of the gastrocnemius and soleus muscles using a cryostat (CM1950; Leica, Wetzlar, Germany) at −25 °C and mounted on amino silane-coated glass slides. mATPase staining was performed using the calcium method to distinguish between type I (slow-twitch) and type II (fast-twitch) muscle fibers. Sections were subjected to mATPase staining following alkaline preincubation at pH 10.7. The sections were then washed with 1% CaCl_2_ and allowed to react with 2% CoCl_2_. Then, they were washed with 0.005 M sodium barbital and distilled water and were subsequently stained with 2% ammonium sulfide. After staining, sections were dehydrated using a graded series of ethanol rinses, immersed in xylene, and embedded in Permount Mounting Medium (Falma Inc., Tokyo, Japan). The stained sections were then observed using a microscope (BZ-X800; Keyence, Osaka, Japan). Approximately, >150 muscle fibers were randomly selected from each section for measurement of the ratio of muscle fiber types. The sample size was set to 4 for each group, for a total of 16 samples.

### 4.9. Immunofluorescence 

Transverse tissue sections (10 µm thickness) were cut from the middle part of the lateral head of the gastrocnemius and soleus muscles using a cryostat (CM1950; Leica, Wetzlar, Germany) at −25 °C and mounted on amino silane-coated glass slides. Subsequently, the transverse sections (10 µm thickness) were fixed in 4% paraformaldehyde and rinsed with phosphate-buffered saline (PBS; pH 7.4). The sections were incubated in PBS containing 1% normal goat serum and 0.3% Triton X-100 at 4 °C for 1 h. The sections were then incubated with mouse monoclonal anti-DHPRα1 antibodies (MA3-920; Thermo Fisher Scientific, Waltham, MA, USA; 1:100) and rabbit polyclonal anti- SERCA1 antibodies (22361-1-AP; Proteintech, Rosemont, IL, USA; 1:500) in PBS containing 0.3% Triton X-100 at 4 °C for 24 h. Subsequently, the sections were incubated with anti-mouse Alexa Fluor 488 Conjugate (A11001, Thermo Fisher Scientific, Waltham, MA, USA) and anti-rabbit Alexa Fluor 555 Conjugate (4413S, Cell Signaling Technology, Danvers, MA, USA) secondary antibodies diluted at 1:1000 in PBS for 1 h at 25 °C. After rinsing with PBS, the sections were embedded in DAPI Fluoromount-G (SouthernBiotech, Birmingham, AL, USA). Thereafter, the stained sections were observed under a microscope (BZ-X800; Keyence, Osaka, Japan).

### 4.10. Statistical Analysis 

KaleidaGraph statistical analysis software version 4.5.1 (Synergy Software, Reading, PA, USA) was used for the statistical analysis. Significant differences between young and old mice were determined using Student’s *t*-test. The means were considered statistically significant at *p* < 0.05. All data are expressed as mean ± standard deviation.

## 5. Conclusions

In this study, we examined the effects of aging on the functionalizing factors of the sarcoplasmic reticulum in mouse skeletal muscle. First, qPCR results showed that the expression of *Atp2a1*, *Atp2a2*, *Sln*, and *Pln* increased with age in the gastrocnemius muscles, but not in the soleus muscles. Subsequently, IHC revealed ectopic SERCA1 and SERCA2a IR only in the gastrocnemius muscles of aged mice. Histochemical and TEM analysis identified TAs, aggregations of the sarcoplasmic reticulum, in the gastrocnemius muscles of aged mice. DHPRα1, RyR1, JPH1, and JPH2, which contribute to sarcoplasmic reticulum function, were also localized in or around the TA using IHC. In addition, JPH1 or JPH2 co-localized with MMP2 around the TA. These results suggest that the sarcoplasmic reticulum as well as functionalizing factors localize in or around the TAs generated in fast-twitch muscles during aging, but that JPH may be degraded by MMP2 around the TA.

## Data Availability

The data presented in this study are available on figshare as follows. SERCA1 localization (Sample #1–8): https://doi.org/10.6084/m9.figshare.25124426, accessed on 3 February 2024. SERCA2a localization (Sample #1–8): https://doi.org/10.6084/m9.figshare.25124462, accessed on 3 February 2024. HE staining (Sample #1–8): https://doi.org/10.6084/m9.figshare.25124471, accessed on 3 February 2024. Modified Gomori’s trichrome staining (Sample #1–8): https://doi.org/10.6084/m9.figshare.25124474, accessed on 3 February 2024. SDH staining (Sample #1–8): https://doi.org/10.6084/m9.figshare.25124480, accessed on 3 February 2024. DHPRα1 localization (Sample #1–8): https://doi.org/10.6084/m9.figshare.25124483, accessed on 3 February 2024. RyR1 localization (Sample #1–8): https://doi.org/10.6084/m9.figshare.25124486, accessed on 3 February 2024. JPH1 localization (Sample #1–8): https://doi.org/10.6084/m9.figshare.25124489, accessed on 3 February 2024. JPH2 localization (Sample #1–8): https://doi.org/10.6084/m9.figshare.25124501, accessed on 3 February 2024. MMP2 localization (Sample #1–8): https://doi.org/10.6084/m9.figshare.25124507, accessed on 3 February 2024. mATPase staining (Sample #1–4): https://doi.org/10.6084/m9.figshare.25124510.v3, accessed on 3 February 2024. DHPRα1 and SERCA1 co-localization (Sample #1–8): https://doi.org/10.6084/m9.figshare.25124519, accessed on 3 February 2024.
